# Death from Drinking Naphtha

**Published:** 1856-11

**Authors:** 


*Death from Drinking Naphtha.*

The *London Lancet* records a case of death from drinking about three ounces of naphtha, used for burning in lamps. The patient was a lad twelve years of age. The symptoms were at first those of excitement, speedily followed by stertorous breathing and a state of collapse. Death took place in less than three hours. At the post mortem examination, the preservative action of the naphtha was very remarkable. The weather was very hot, and although three days had elapsed since death, all parts of the corpse were as fresh as if the lad had recently died. The blood was everywhere very fluid. The lungs were not at all congested, and the coats of the stomach were found to be very little affected by the presence of the poison. The smell of naphtha pervaded the whole of the tissues, and was very perceptible immediately on opening the head.

